# The life and death of confidentiality: a historical analysis of the flows of patient information

**DOI:** 10.1057/s41292-021-00269-x

**Published:** 2022-01-29

**Authors:** Sarah Wadmann, Mette Hartlev, Klaus Hoeyer

**Affiliations:** 1 The Danish Center for Social Science Research – VIVE, Herluf Trolles Gade 11, 1052 Copenhagen, Denmark; 2 Centre for Legal Studies in Welfare and Market – WELMA, Faculty of Law, University of Copenhagen, Copenhagen, Denmark; 3 The Centre for Medical Science and Technology Studies – MeST, Department of Public Health, University of Copenhagen, Copenhagen, Denmark

**Keywords:** Confidentiality, Datafication, Digitalization, Information technology, Secrecy, Zombie categories

## Abstract

Health data can contain sensitive information. People who consult a doctor seek help on issues that matter to them: they typically expect some form of confidentiality. However, the notion and practices of confidentiality have changed dramatically over time. In this article, we trace the history of confidentiality in the Danish healthcare system, which has one of the world’s most integrated patient information infrastructures. Building on an analysis of legal and political documents dating back to the late seventeenth century, we show that confidentiality originated as a social phenomenon that helped build trust in healthcare professionals and gradually developed into an idiom of citizens rights. Lately, confidentiality has given way to more technocratic forms of data protection. As the political, legal and technological reality, which the idea of confidentiality once referred to, has radically changed, we argue that confidentiality has become what Ulrik Beck has called a ‘zombie category’—a notion that lives on even if its content has passed away. If confidentiality has become a zombie concept, we suggest it is time to discuss what may take its place so that patient interests are protected in the current political economy of health data.

## Introduction

There’s like a boundary, you see,” Bente explains and points to the drawing between us. Bente is a general practitioner (GP) who agreed to be interviewed about information flows in Danish general practice. In the middle of the paper, Bente has circled a physician and a patient in a relationship. The circle, she suggests, marks the boundaries of a confidential space. “What goes on in this space is secret”, she says and points to the circle. “The two of us [physician and patient] decide what can be shared with others!” Pausing a moment, Bente then adds: “It’s just not how we work anymore. (Interview and fieldnotes, June 2016).^1^

We had approached Bente for an interview because she was, and is, a vocal critic of sharing GP’s medical records on national, digital platforms. We wanted to understand why. Bente was concerned about a revision of the legal framework that obliges her to use diagnostic codes and report patient information to a national database, and asked with agitation pointing to her drawing: “What is it [the politicians] want in here, (…) in this secret space?” We asked her to map out which pieces of patient information that flow from GP clinics to various other parties. The drawing quickly turned into a complex water pipe-like structure connecting the allegedly secret space with a plethora of other actors in an ever-expanding infrastructure. This interview was one of the first times in the course of a larger project about data-intensive medicine (see https://www.policyaid.ku.dk) when we realized that a discrepancy had emerged between how health professionals talk about confidentiality, and how contemporary information infrastructures work in practice. We subsequently asked for similar drawings from other health professionals who did not consider themselves critical towards information sharing. And we noticed their own surprise as they mapped out the data flows from what they otherwise spoke of as a confidential space. It is not uncommon, apparently, for health professionals to experience a disconnect between the perception of the medical encounter as a confidential space between people, on the one hand, and the technologically mediated and politically governed flow of patient information, on the other. How can we understand this apparent disconnect? How did it arise? How have the political, legal and technological means of restricting and facilitating information flows changed over time and what are the implications for confidentiality?

In this article, we investigate how the flow of patient information has been shaped over time in a political economy operating through the interaction between politics, law and technological innovation (Prainsack 2020). We use information as an overarching concept and define patient information broadly as spoken, written or visual messages or diagnostic codes pertaining to a person who seeks help for a health-related issue. Patients may generate and exchange such information in many contexts, from Facebook to commercial genetic testing companies (Gross and Geiger 2021; Henwood et al. 2003), but we focus here on flows of patient information attributable to activities in the healthcare system.

We draw on a historical analysis of the Danish healthcare system, which constitutes an informative case for investigating the role of confidentiality through the transition to a still more data-intensive type of healthcare. Denmark has one of the most advanced and integrated information infrastructures in the world, and a very liberal regulation of the repurposing of patient information for government statistics and research. The assignment of a unique, trackable identification number to each citizen makes it possible to follow citizens’ interactions with government services throughout (and beyond) the course of their individual lives. This has facilitated extensive integration of information about patients and citizens at the national level (Bauer 2014). With significant public investments, Denmark has pioneered the digitalization of medical records and the development of shared communication standards that enable data exchange across providers (Kierkegaard 2011, 2013). As a technological frontrunner, this development in Denmark anticipates many of the opportunities and challenges that the rest of Europe is likely to face when embarking on investments in integrated information infrastructures, such as the European Health Data Space or initiatives like smart4health (www.smart4health.eu).

We suggest that information flows not only rely on political, legal and technological means to *facilitate* information exchange, but also on efforts to *restrict* such flows. Non-flows are constitutive of flows, because they facilitate the confidence patients need to be willing to share sensitive information. The analysis shows that attempts to restrict the flow of patient information have a long legacy. Yet ideas about desirable and non-desirable information flows have changed substantially over time. Before the 1930s, informal chatter was seen as the main risk in the management of patient information, and secrecy was nurtured as a virtue for individual health practitioners. As we will show, ideas about informational risks have shifted gradually over time, as patient information transformed from oral stories, to paper-based documentation, and later, to digital data. In the meantime, professional assurances of secrecy turned into regulatory ideals of confidentiality, which have now given way to data protection as a more technical and rule-based regime. At a time when patients are sometimes considered sources of value in expanding data markets, the history of confidentiality serves as a reminder of the importance of respecting patient interests and regulating the flow of sensitive information. Still, the notion of confidentiality appears to be of little help in upholding patient interests in data-intensive healthcare. References to ‘confidentiality’ nevertheless linger on in policy documents and social expectations, but the phenomenon it used to refer to seems to have disappeared. Political, legal and technological changes have rendered the original meanings of the term obsolete, because on contemporary digital platforms of health data, health profesionals no longer control the patient information they produce. Ulrik Beck has suggested the term ‘zombie category’ for concepts that live on even when the social forms they referred to have passed away (Beck and Beck-Gernsheim 2002, p. 201–13; Beck and Willms 2004, p. 51–2). If we may have to consider confidentiality a zombie category, what should take the place of ‘confidentiality’ in caring for patient interests?

We start by situating our analysis in social science theories about confidentiality and secrecy, and outline how we investigate the interplay between politics, law, and technology. Then, we describe our methodological approach. The subsequent analysis is structured into four parts. Each part describes a historical period characterized by political, regulatory and technological transformations that enabled the notion of ‘confidentiality’ to acquire a life and gradually become a living dead.

## Confidentiality at the intersection of politics, law and technology

Many of the problems that make patients go to see a doctor are delicate ones. They involve confiding potentially embarrassing pieces of information to a professional who might be a complete stranger. Geissler (2013) and Jones (2014) have argued that what counts as sensitive information differs between individuals and varies between societies. Surveys among European populations suggest that issues related to sexuality, substance abuse, and mental health are typically considered particularly sensitive (Larsen et al. 2019). To confide such information to others not only presupposes a particular form of relationship—it generates it. To reveal intimate information involves an intricate social dynamic between people (Fainzang 2002). Simmel summarized this in his seminal analysis of secrets:

the trust we *receive* contains an almost compulsory power, and to betray it requires thoroughly positive meanness. By contrast, confidence is ‘given’; it cannot be *requested* in the same manner in which we are requested to honour it, once we are its recipients (Simmel 1950a, p. 348).

The handling of patient information matters to—and works upon—those who need to confide in health professionals, as well as those professionals who come to act as guardians of other people’s secrets. ‘Secrecy’ refers to a social expectation of unconditional withholding of information in a relationship between people. ‘Confidentiality’ also pertains to a relationship between people, but the withholding of information is not unconditional. Rather, confidentiality refers to a regulated flow of information: information is conveyed to others only according to an agreement, or to predefined rules. These rules can be compared to what Nissenbaum has described as tacit and “context-relative informational norms” relating to actors (who receives information), attributes (types of information), and transmission principles (constraints on flows) (Nissenbaum 2010, pp.140–147). As long as these rules correspond to patient expectations, a transfer of information—e.g. among health professionals—is not privacy infringement.^2^ Confidentiality implies that the health professional in whom the patient confides curates the information, which is to be passed on. Curation, in this sense, refers to a “discriminate selection” of information in order to control what is shared with whom (Davis 2017, p. 773). Johansen and Andrews (2016) have suggested that without such curation, patients might not feel confident to entrust professionals the information that is needed to identify the correct diagnosis and commence adequate treatment.

The ability of health professionals to control the flow of patient information has changed over time. Nearly fifty years ago—at a time when doctors were mostly men and technological options for information sharing were more limited—Grossman (1977) pointed to how the political ambition of mitigating societal risks interacted with legal requirements, and prompted doctors to consider how to balance a concern for confidentiality with the duties of reporting:

For his (sic) patients’ sake and increasingly for his own as well, the physician would be well advised to learn the narrow but tortuous path between the edict ‘reveal not your patients’ data’ and growing demands that he do just that (Grossman 1977, p. 43).

Since then, information generation, storage and exchange has changed radically along with the ever more pervasive digitalization and the changing political and legal frameworks. It is through these changes in the political, legal and technological conditions for information storage and exchange that we identify the ‘life and death’ of confidentiality.

We conceive of politics broadly as the “relations between all the institutions, which together ‘govern’ social, economic and political life in a society” (Leftwich 2004 (1984), p. 15). This notion of politics is not limited to the formal activities of government, or the ideals and concerns expressed by politicians, but includes also the attempts of other parties to gain influence through formal or informal mechanisms. Politics interacts in various ways with law and technology to enable a realization of political ideals (Jasanoff 2007). Flows—and non-flows—of patient information are shaped by their interaction. Legal regulation may be used by political decision-makers to address particular political concerns or visions, or to legalize already existing practices. However, legal regulation may also shape political conceptions of what is worrisome or worth pursuing, as legal documents encode particular understandings of the phenomenon that is subject to regulation (Hurlbut et al. 2020). In our analysis, we seek to understand this double capacity of legal regulation: to both enable a realization of political ideals, and to shape ideas about the risks that are to be mitigated.

Information is always embodied and mediated: it is co-produced with the media through which we encounter it. Information scholar O’Riordan writes: “Information, like humanity, cannot exist apart from the embodiment that brings it into being as a material entity in the world” (2017, p. 127). The introduction of record-keeping systems comprised of individual index cards and, later, punchcard technologies, for instance, allowed more flexible sorting, aggregation and stratification of information (Mattern 2020). Koopman et al. (2021) describe how the media of medical record keeping ‘format’ information, so that administrative and fiscal information requirements come to shape the formats used in the clinic, which in turn can affect clinical practices (Koopman et al. 2021). Formats matter: paper records, punchcards and digital platforms all have different affordances. Once clinical information is accesible for public administrators, it also allows them to know about, and govern, populations in new ways (Bauer 2014). When information is stored in an electronic format on integrated networks, it is both searchable and computable. The information has become data. Data can be letters, codes or numbers, but they are always searchable and subject to some form of computational processing. Datafication therefore affects conditions of confidentiality. In the analysis, we thus pay analytical attention to how emerging information technologies influence—but do not determine—the flow of patient information.

Technological change also matters for what may be seen as the major threats to the confidential treatment of patient information, and what to do about it. When information changes from oral to written form, for example, new practices are needed to keep it secret. The very existence of a document makes the revelation to third parties a potential risk (Simmel 1950b) calling for various archiving practices. Bowker suggests that the emergence of electronic databases marks a new “epoch of potential memory” where –

the question is not what the state ‘knows’ about a particular individual, say, but what it can know *should the need ever arise* (Bowker 2005, p. 30, italics in original).

Bowker alludes to a transformation where ‘knowing’ is not about personal acquaintance, or the communication of a narrative about another person, but the possibility of reconstructing an account of a person through the integration of data points that may be scattered over multiple collections. In contrast to the telling of a secret to somebody, the potential recipients of sensitive information are not yet known and the results of the data integration (e.g. a risk prediction) may even be unknown to the individual it concerns.

Some types of informational exchange go beyond ‘relations-between-people’ and refer to systems and algorithmic types of information exchange; what Amoore (2019) refers to as “post-human knowledge”. In post-human knowledge, the ‘knower’ might be a computer rather than a person. While the confidential treatment of patient information tended to be about ‘who knows what about whom’, it is now often a matter of ‘what knows what about whom’. Automated and computer-mediated information storage and exchange creates radically different conditions for the treatment of sensitive patient information (Crawford 2021). Confidentiality is consequently eroded as a virtue that emerges in the relationship between people. Still, even without a human ‘knower’, the exchange of sensitive information can affect people through automated forms of decision-making based on data circulating about them (Pasquale 2015). The use of health information for purposes beyond clinical care can interact with patient identities and have an emotional impact (Cheney-Lippold 2017; Ebeling 2016). It affects the conditions under which people seek help when confronted with illness, and the bargain they must strike between privacy and care (Petersen 2019). In summary, to understand the rise of contemporary concerns of doctors like Bente, we investigate how politics, law and technology interacted over time to shape the flow of patient information.

## Materials and methods

The empirical material which informs the historical analysis consists mainly of legal and political documents. The original impetus, however, comes from a series of interviews with politicians, patients, healthcare managers and health professionals carried out by Sarah Wadmann and Klaus Hoeyer between 2014 and 2021. The interviews, including our conversation with Bente, alerted us to a disconnect between a professional sense of a duty of confidentiality, and the contemporary data integration that characterizes the healthcare system in which the professionals work. To better understand the origin of this disconnect, we started tracing the political, legal and technological history of the informational requirements, rights and ideals in Danish healthcare with legal scholar Mette Hartlev. We read existing historical analyses of the Danish healthcare system and retrieved government white papers, law proposals, legal acts and other national documents that were referred to as influential markers of political or regulatory change in existing analyses. An online database of legal acts (Retsinformation.dk) enabled us to systematically trace legal changes over time and retrieve legal acts, including legislative preparatory work. Appendix 1 provides an overview of the historical documents. Through the documents, we identified shifting political ideals and concerns mobilized to justify particular regulatory measures. Our description of technological changes relies mainly on the accounts of technological challenges and solutions provided in the government white papers and secondary analyses of the Danish information infrastructure.

In a broad historical perspective, from a time period with no formal healthcare system and no written records, to the contemporary digital data infrastructures of the Danish welfate state in the 2020s, some obvious changes have taken place. But when, and how, did these shifts come about and what happened to ideas about confidentiality in the process? To identify the gradual shifts, we extracted material from the documents guided by the research questions in Table 1. The research questions were derived from our theoretical framework, and were later refined through our confrontation with the empirical material.

**Table 1:** Research questions

Theme	Research questions
Politics	Which rationales and values do political decision-makers mobilize to advance or restrict the flow of patient information?
	Which risks do policymakers see as in need of monitoring and mitigation?
Law	Which legal solutions are used to (enable and) restrict the flow of patient information?
	Who is considered to be responsible for restricting the flow of patient information?
Technology	Which technologies have emerged and how have they facilitated and restricted the generation, storage and exchange of patient information?
	How does the form of patient information change during the period?

We identified four periods through a dialectical process, where we moved between empirical material, research questions, and the identification of political, legal and technological changes. Recognizing that historical development is “always a mix of continuity and change” (Sewell 2005, p. 9), we were not looking for clear-cut periods characterized by one particular political, legal or technological formation. Political, legal and technological transformations are incremental processes. We therefore searched for breaking points, when political, legal and technological transformations had become obvious, and used these to identify the periods over which the changes had taken place. Accordingly, there are overlaps and continuities among the four periods, but the juxtapositions help to highlight the shifts in emphasis over time. We begin with the emergence of a healthcare system and the earliest legal regulation of confidentiality (-1930s). Then we explore the expansion of the welfare state while moving from paper to computer (1930–1967). Then a shift to simple digitization (1967–2000). And finally to advanced infrastructural integration, including platform organization, new types of data pooling, and multiplication of the purposes of data use (2000-). An overview of the periods, and the shifts they involve, can be found in Table 2.

**Table 2:** Summary of political, legal and technological transformations in the four periods

Transformation/periods	Before 1930	1930–1967	1968–1999	2000 onwards
**Politics**				
Rationales and values mobilized to advance the flow of patient information	Monitor risks to society	- Monitor risks to society - Remuneration and administration - Collaboration between professionals - Research	- Monitor risks to society - Remuneration and administration - Collaboration between units - Research - Accountability	- Monitor risks to society - Remuneration and administration - Collaboration between sectors - Research - Accountability - Economic growth
Perceived risks to be monitored and mitigated	Gossip	Unauthorized disclosure	Information integration	Data leakage and hacking
**Law**				
Legal solutions used to enable and restrict information flows	Promises of secrecy	Professional duties of confidentiality	Citizen rights and organizational duties	Data protection
Actors considered in control of restricting the flow of information	The individual professional	The profession	Government authorities and patients	Institutional data owners
**Technology**				
Emerging information technologies and forms of patient information	Orally conveyed stories	Written documentation stored in paper archives	Digital data stored in separate electronic registries	Digital data stored in electronic platforms that integrate multiple information sources
**Confidentiality**				
Conceptualization of restricted information flows	Secrecy (individual relations)	Confidentiality (within the professional relations)	Confidentiality (within the healthcare system)	Data protection (health data ecosystem of public and private actors)

## Secrecy as a moral virtue: Interpersonal exchange of patient stories (before the 1930s)

Early documents about patient information convey that promises of secrecy were the primary means by which health practitioners established themselves as trustworthy persons whom patients could confide in. At a time when patient information was mainly exchanged orally, and rarely documented, secrecy signalled a moral quality. Practitioners should possess this moral quality to rise up above gossip and honour and sustain the relationship with the patient. We develop this in the following.

The earliest documents about patient information that we have been able to identify are a set of royal executive decrees on pharmacists and midwives. In 1672, a royal decree instructed pharmacists not to “enunciate or reveal” information about the medical condition of patients unless concealment might lead to danger (Forordning om Medicis oc Apotecker 1672, § 24). A decree from 1750 established a particularly strong commitment to secrecy on midwives working at the Royal Birth Clinic (Kgl. reskript af 13. marts 1750; Kgl. reskript af 6. januar 1764; Instruks for alle til praksis berettigede jordemødre 1896). The Royal Birth Clinic was a place where women could give birth in secret (the so-called clandestine birth).

In 1815, an ethical obligation of secrecy for medical doctors in Denmark was introduced through a medical oath (Kancelliskrivelse af 15. august 1815). This oath was aligned with the Hippocratic Oath, originating in Ancient Greece, in which secrecy was considered a moral quality of the doctor: “And whatsoever I shall see or hear in the course of my profession, as well as outside my profession in my intercourse with men (sic), if it be what should not be published abroad, I will never divulge, holding such things to be holy secrets” (from translation in Wikipedia). In an article published in a Danish legal journal in 1905 that reflected on this obligation, it was stated that medical doctors were expected to demonstrate “tactfulness and act with propriety” in relation to patient information (Olrik 1905, p. 198). The reference to tactfulness and propriety implied that secrecy was understood as a token of interpersonal respect and good manners. Moreover, secrecy was depicted as a precondition for medical practice:

On the one hand, no information that is relevant for the treatment of disease should be withheld from them [the medical doctors], - on the other hand, those who confide in the doctor should not risk to see their secrets relinquished (Olrik 1905, p. 206).

To create a relationship where patients were assured enough to reveal sufficient information for the doctor to assess their condition, secrecy constituted a “shield” so that the information “would not be used arbitrarily outside the particular purpose for which the confidence is shown” (Olrik 1905, p. 207). In line with Simmel’s (1950a) analysis of secrecy, these early regulations expressed an understanding of patient information as personal secrets and conveyed an expectation of health professionals to honour and sustain the interpersonal relationship in which these secrets were revealed.

The conception of secrecy as a virtue to be nourished by individual practitioners reflects a tradition of oral and interpersonal information exchange. In the seventeenth and eighteenth century, there was no statutory duty to keep medical records. However, the demand for written documentation increased towards the end of the period (i.e. in the early twentieth century). This change was linked to technological innovations—including new record systems—but also reflected new social policies, medical practices and legal frameworks. The public provision of social care for those unable to support themselves was introduced with the Danish 1849 constitution. The poverty act was revised in 1891 to ensure access to public subsidies for doctor and midwife services as well as burials, and in 1892, a public health security act (Sygekasseloven 1892) introduced a voluntary, insurance-based healthcare scheme. This emerging system of public sector support required archival and communication technologies that went beyond human memory and oral narratives. Government interests in keeping population records at the municipal level and, later, the national level required standardized information (Bauer 2014). From 1914, midwives were required to keep structured records and provide standardized reports of all births and abortions due to ‘societal interests’ in keeping track of the population (Jordemoderloven 1914). A shift from parish lists and vital statistics tables, to a system of individual index cards by the end of the 1920s, enabled more flexible record keeping in the public administration, and a quicker generation of lists, e.g. for welfare recipients (Bauer 2014). As symbols of social, economic and technological progress, such filing systems were even exhibited at the World Fairs from the late nineteenth century onwards (Mattern 2020).

The understanding of secrecy as a moral virtue implied that health practitioners were to judge on a case-to-case basis whether the keeping of one person’s secret might cause danger to other people and to weigh these concerns. In the legal discussion about the secrecy of medical doctors from 1905, it was stated that doctors were expected to reveal information about patients in some situations—for instance if a doctor learned about a case of child battering or realized that persons suffering from mental illness or infectious diseases (such as tuberculosis or venereal diseases) posed a risk to others (Olrik 1905). Such a trade-off between secrecy and danger was even expressed in the early regulations concerning pharmacists (Forordning om Medicis oc Apotecker 1672, § 24) and midwives (Instruks for alle til praksis berettigede jordemødre 1896). Hence, the individual practitioner had to balance the ideal of secrecy against a concern for risks to society.

In summary, to encourage the flow of information from patients to health practitioners, secrecy was understood as a moral virtue to be nourished by individual practitioners. Yet individual health practitioners were also expected to exert judgement and reveal information about individual patients when deeming them a medical or moral risk to the population. The gradual introduction of written patient information introduced new risks of revelation (cf. Simmel 1950b) and paved the way for more regulated transfers of patient information. In the course of these developments, virtues of secrecy turned into professional duties of confidentiality. This is what we outline in the next section.

## Confidentiality as a professional duty: Expanding archives of written documentation (1930–1967)

By the 1930s, the understanding of secrecy as a moral virtue had given way to a statutory duty of confidentiality. The policy ambition was to establish health practitioners as members of trustworthy professions whom patients could speak to in confidence, while simultaneously ensuring that these professions served the administrative and economic interests of the expanding welfare state, which now paid for their services.

A series of legal reforms regulated the rights and obligations of professional groups in the early twentieth century. These reforms specified the professional duties of confidentiality. The first reforms concerned dentists, pharmacists and midwives (Lov om Apotekervæsenet 1913; Jordemoderloven 1914; Lov om udøvelse af tandlægegerning 1916), and from the early 1930s, elaborate laws were enacted that defined the responsibilities of nurses and medical doctors (Lov om autoriserede sygeplejersker 1933; Lov om udøvelse af lægegerning 1934). For medical doctors, the duty of confidentiality implied that they could be held accountable for the disclosure of patient information to “unauthorized persons”. This was to ensure that “the information entrusted to the professionals will not be used arbitrarily outside the particular purpose for which they are intended” (Ministry of Interior 1931, p. 27). Notice the notion of ‘unauthorized’: it was now clearly possible to imagine an *authorized* transfer of information, which is something substantially different from the individual discretion expected in the earlier period. In a government white paper, the duty of confidentiality for medical doctors was motivated by a dual concern for individuals and for the population’s health:

If medical doctors were entitled to speak of everything they might learn about the illnesses or other circumstances of their patients, it could be feared that people, for this reason, would refrain from consulting a doctor or omit information. This would be contrary to the interests of the individual as well as to public hygiene (Ministry of Interior 1931, p. 27).

Just like in the previous period, the confidential handling of patient information was considered a precondition for patients to seek medical attention and convey information. Self-taught practitioners were still popular in the 1930s, and there was political concern that the “unskilled treatment” offered by “quacks” would work against public health efforts of infection control and levy “economic burdens” on “the public administration or the insurance” (Ministry of Interior 1931, p. 13).

In the 1930s, the ideals of social justice that emerged in the nineteenth century evolved into a political programme to establish a redistributive welfare state (Jacobsen and Larsen 2017, pp. 328–329). Major social reforms transformed social services from alms that were targeted the poor, to a right of citizens (Fridericia 1934). These transformations also impacted the conception of, and flow of, patient information. While patient information was previously conceived as secrets that could be revealed and protected within a personal relationship, it was now seen as written documentation exchanged among patients, health professionals and the institutions of the welfare state**.** To ensure impartiality and fairness in the distribution of welfare services, government authorities increasingly requested “objective” and “unbiased” information from health professionals to document the entitlements of citizens (Ministry of Interior 1931, p. 43). From 1937, medical doctors were obliged to keep medical records to document cases of diseases and accidents (Bekendtgørelse om lægers pligt til at føre optegnelser 1937) and provide a variety of “declarations for public use” (Backer and Skovgaard 1949, p. 86). Administrative demands for patient information were also fuelled by a system of cost equalization among the local governments that ran the hospitals (Lov om sygehusvæsenet 1946, §8), and attempts to identify ‘excessive use’ of government-subsidized health services (Lov om folkeforsikring 1953, §27).

In addition to the welfare state programme, demands for written documentation arose from a need to facilitate interprofessional communication at a time of increasing medical specialization (Ministry of Interior 1931, p. 83) and expanding medical research (Bauer 2014). In the 1940s, the first national registries were set up to provide for medical research, including the Danish Cancer Registry in 1942, and the Twin Registry in 1954 (Ministry of Justice 1976, p. 116). The expanding requirements to document clinical work, and to report to administrative systems and registries, gave way to a new professional group: medical secretaries entered Danish hospitals in the late 1930s (Hollmann 1989). The information technologies used by the secretaries and other professionals were still analogue: typewriters, index cards, filing cabinets and pre-printed forms dominated the practices of information generation, storage and exchange (Hollmann 1989; Bertelsen et al. 2021).

These information flows were embedded in particular power relations. While patient information was exchanged among health professionals and government authorities, patients had no access to these informational archives. To foster “candid statements” from the professionals and “protect” the patient from distressing information, patients’ access to their own medical information was very limited (Backer and Skovgaard 1949, pp. 87–88). Patients had no legal right to be informed about their condition or to have access to the records that were kept about them. It was up to the health professionals to decide which information to reveal (Hartlev 2013, pp. 43, 173). Medical certificates could also be kept secret from patients to facilitate the passing on of “objective and unbiased information” from medical professionals to government authorities (Ministry of Interior 1931, pp. 30–31, 43). As in the previous period, the professionals had to exert judgement, but now the judgement appeared to revolve mainly around whether, or when, to inform *patients.* Confidentiality had emerged as a duty embedded in a relationship of power where professionals—not patients—were seen as the proper guardians of patient information.

To sum up, the political ambition of creating a fair and redistributive welfare state generated informational requirements. These requirements extended the flow of patient information from health professionals to government authorities—and at the same time stopped patients from accessing this information. Professionals had to increasingly balance a concern for confidentiality against the administrative and economic interests of the welfare state. While the generation and exchange of patient information relied on written documentation stored in paper archives, electronic data processing systems were introduced by the end of the 1960s to deal with the growing amount of information. At the World Fair in 1964, it was no longer analogue technologies like filing cabinets and cataloguing systems that caught people’s attention, but the IBM pavillion that displayed a digital age to come (Mattern 2020).

## Confidentiality as citizens' rights and organizational duties: digitization of patient information (1968–1999)

From the mid-1960s, ideals of rationalization dominated the political debate on information technologies. They paved the way for digital solutions that gradually transformed patient information into data formats and enabled a vast expansion of the national information infrastructure. With increasing political and legal attention to citizens rights, the concept of confidentiality changed also. Confidentiality turned into a matter of citizens rights and organizational duties, as we demonstrate in the following.

The personal identification number (CPR) was introduced in 1968, and became the backbone of an ever more encompassing information infrastructure. The CPR registry was a simple registry for the authentication of citizens, and at the time it was considered a “routine matter of technical modernization” (Bauer 2014, p. 192). The registry provided all citizens with a unique identifier to be used in all interactions with public authorities and, thereby, enabled a linkage of personal information from ever more registries. By 1976, it was estimated that about 500 electronic registries existed in Denmark with an annual growth of about 50 registries (Ministry of Justice 1976, pp. 62–63), each of them using the CPR as a reference number for all registrations. This extension of the national information infrastructure was facilitated by public investments in electronic data processing systems. In the largest hospitals, the introduction of systems like “the Electro Brain IBM 1800” (Elektronhjernen) in the late 1960–1970s signified a new epoch in the processing of patient information (Ministry of Justice 1976, p. 104; NN 1967) and local governments increasingly invested in electronic systems to process medical and administrative information (Ministry of Interior 1977). In government white papers, these investments were described as a precondition for efficient planning, productivity and cost control (e.g. Ministry of Interior 1977).

In parallel with this heralding of technological optimization, a political concern emerged that the technological developments also involved risks to be monitored and mitigated: the “agility and ability [of electronic systems] to process enormous amounts of data constitute a benefit as well as a risk” (Ministry of Justice 1976, p. 18). The envisaged risks no longer concerned human information ‘leaks’, as in previous periods, but the ability to assemble and combine digital information from various sources. A government white paper listed risks such as the “unwanted mass use of personal information” for “selective advertising” or “social manipulation”, “unnecessary data accumulation” and the creation of “integrated data banks” through the linkage of data sources (Ministry of Justice 1976, pp. 13, 15–16). In addition, a risk of “data exodus” [Danish: dataflugt] was mentioned, i.e. the transfer of sensitive data across national borders enabled by extended “telenetworks” and other technological solutions (Ministry of Justice 1976, p. 43). There was a call for supra-national regulation to avoid some countries turning into a “data paradise” for private enterprises seeking “great data power” and liberal regulation (Ministry of Justice 1976, p. 43). It is striking to read these concerns in the 2020s, as they were a premonition of the global regimes of digital surveillance that were to come into being only decades later.

To safeguard the use of the information, the retrieval and linkage of register information required explicit permission from government authorities (Retsudvalget 1978). In 1978, two pieces of legislation were adopted that regulated the electronic collection, storage and use of personal data (Retsudvalget 1978; Registerudvalget 1973).^3^ Following a political decision, CPR information would not be provided to private sector institutions (Ministry of Justice 1976, p. 82). However, in pace with digitization, public institutions came to lack the capacity for information storage and processing, and the operation of national registries was outsourced to specialized enterprises (Ministry of Justice 1976, pp. 62, 66). Later, the biggest of these enterprises have been privatized and are now owned by private equity funds (Hoeyer 2020). It became increasingly difficult to distinguish between public and private actors in the regulation of information flows. Instead, a practice evolved where the reuse of register data was considered legitimate by government authorities if it was congruent with a “public purpose”—while “mercantile or rationalization interests” were still considered illegitimate (Ministry of Justice 1976, pp. 82–83).

At the same time, the power relations between health professionals and patients changed, and the regulation of information flows came to focus on the newly emerging citizens’ rights. A special provision on the disclosure of personal data was inspired by the European human rights movement’s attention to citizens’ rights to private life from the late 1960s onwards (Ministry of Justice 1976, p. 13).^4^ The provision entered into force in 1985 with profound implications for the disclosure of patient information, which now came to require written, informed consent in many situations (Ministry of Justice 1984; Forvaltningsloven 1985). This principle of asking for explicit permission from patients before disclosing information was consolidated with the adoption of legislation on patients’ rights in 1998 (though this legislation also provided for more effective communication across healthcare providers) (Patientretsstillingsloven 1998). The political focus on citizen rights also meant that it was no longer considered appropriate to conceal personal information from patients. Citizens acquired a right to know which information was kept about them in public registries in 1978 (Retsudvalget 1978), and from 1987 to 1994, legislation was passed that gradually extended patients’ rights of access to the information in their medical records (Lov om offentlighed i forvaltningen 1987; Lov om ændring af lov om private registre m.v. og lov om offentlige myndigheders registre 1987; Lov om aktindsigt i helbredsoplysninger 1993). The regulatory ideal of confidentiality was changing: the information transfers within the fast-expanding infrastructure were now subject to patient scrutiny, and confidentiality turned into regulated organizational practices, rather than a matter of professional judgement.

Meanwhile, the calls for improved technological solutions intensified. The magnetic tape technology used by most public registries and information systems in the beginning of the period only allowed for periodical information updates (Ministry of Justice 1976, p. 86). The resulting delays meant that the registered information was of limited use in day-to-day patient care and administrative case handling (Ministry of Justice 1976, p. 104). Likewise, researchers had to manually retrieve and combine different databases when they wanted to use information from the public registries, which was time consuming. Calls for “real-time data processing” emerged in the late 1970s as a result (Ministry of Justice 1976, pp. 104–106). As a forerunner of the digital platforms that were to come, some hospitals experimented with online hospital information systems already in the late 1970s to allow for more speedy and flexible data processing (Ministry of Justice 1976, pp. 104–106).

To sum up, the changes in political ideals, legal regulations, and information technologies, all transformed how confidentiality was practiced. The digitization of public registries and hospital information systems from the late 1960s meant that patient information was increasingly turned into digital data that could be linked and used for multiple purposes. Individual patients and government authorities were expected to act as gatekeepers and control the reuse of patient information.

## Data protection as technocratic rules: digital platforms and automated data pooling (from 2000 onwards)

At the turn of the millenium, patient information was almost fully digitized in Denmark, and political attention turned to the use of digitalization as a motor for societal change. Reflecting the shift to computers operating in integrated networks as the primary medium for conveying patient information, most regulatory discourses came to focus on digital data. While health professionals continue to talk about confidentiality, the professional *duty* of confidentiality has lost its practical importance. The regulation of information flows now centers on data protection as a set of technocratic rules. We develop this shift in the following.

In contrast to the previous period, where data linkage was seen as a major risk, many reform initiatives came to focus on the integration of data sources. The former political concerns about ‘data exodus’ and the ‘mercantile’ use of patient information have been replaced by a rhetoric where cross-border data transfer is pictured as a driver of value creation. Regulators now tend to focus on the lack of data access as a main problem to be solved. Meanwhile, the former duty of professionals and government institutions to protect the interests of citizens, has given way to an obligation to empower citizens and provide them with the tools to protect themselves. This political rhetoric is reflected in initiatives taken by the European Union (EU) to create a shared market for information, with the directive for data protection (Council Directive 95/46/EC 1995, in force from 2000 to 2018) and, later, the General Data Protection Regulation (GDPR) (in force since 2018) (Council Regulation 2016/976 2016). An EU data strategy was adopted in 2020 with the goal to “enable Europe to become the most attractive, secure and dynamic data-agile economy in the world” (European Commission 2020a). In this effort, measures are taken to standardize electronic exchange formats to foster the “interoperability of health data” (European Commission 2020a, p. 30). Likewise, a common European approach to artificial intelligence (AI) has been motivated by a need to “reach sufficient scale and avoid the fragmentation of the single market” (European Commission 2020b, p. 2). The strategy aligns with ambitions also found in the Open Data movement. As an EU member state, Denmark has adopted these frameworks. Furthermore, Denmark actively uses patient data to attract interest from the international life science and tech industries. The concerns of the 1970s appear to have become the business strategies adopted in the 2020s (Regeringen 2021, p. 21; see also Tupasela 2017, 2021).

What do policymakers identify as the main risks to citizens in this period and which regulatory measures are used to deal with them? The preamble of the GDPR lists various risks related to big data analytics and AI-based predictions, including discrimination, profiling, reputational damage, financial loss and identity theft (Council Regulation 2016/976 2016). Loss of confidentiality is still mentioned as a risk in legal and political documents (e.g. Council Regulation 2016/976 2016; Ministry of Justice 2017a). However, the proposed solutions to deal with this risk have changed considerably. In contrast to the emphasis on the human curation of information transfers that was integral to the idea of confidentialty, regulatory solutions now focus on *data protection*. As a technocratic solution, data protection implies a focus on citizens rights and technical safeguards. Whereas health professionals were expected to exert judgement and balance concerns in earlier periods, they now increasingly have to follow rules. The GDPR, for example, defines the institutional responsibilities of risk monitoring and of ensuring safety by design, and states that any secondary use of personal data must be compatible with the original purpose of data generation (Ministry of Justice 2017b, § 4). However, exemptions can be justified based on national law or if citizens have consented to the reuse (Databeskyttelsesloven, § 5 2018). It is still not well-established how these rules are to be interpreted in Danish law (Blume 2018; Motzfeldt 2019). In contrast to the informational citizens’ rights introduced in the previous period, the GDPR places little emphasis on informed consent as a legal basis for data processing (European Data Protection Board 2020; Dove and Chen 2020; Article 29 Working Party 2018, p. 4). Rather, citizen rights as specified in the GDPR concentrate on transparency and access, data portability, and the erasure or rectification of incorrect information (Council Regulation 2016/976 2016).

These political and regulatory developments have interacted with technological innovations. Whereas patient information was previously sent to central registries as curated transfers by health professionals, information flows are increasingly automated. Health professionals work on digital platforms, and their medical records are subject to automated transfer and pooling of patient data. The use of prescription servers for pharmaceutical dispensation is an example of a national platform organization. This implies that health professionals (e.g. medical doctors, pharmacists, municipal care workers) as well as patients *log in* to the same centralized platform to enter or change information about a patient’s medication. Health professionals no longer ‘send’ information; they work on servers that are already integrated with centralized databases. The automated pooling of patient data occurs for example in databases used for quality assurance and, since 2003, patients have been able to access information from their medical records online through a national platform (Sundhed.dk), which integrates information from multiple specialist healthcare providers (Frost and Sullivan 2017). In contrast to the power once held by professionals to control access to patient information, they now have very limited ability to control the flow of patient information. And their own retrieval of patient information is logged and can be monitored by government authorities (Sundhedsdatastyrelsen 2021).

Meanwhile, patients increasingly produce and share information about their health through other parts of the platform economy: they order online tests and commercial genetic tests, monitor their health with various kinds of devices, and share the generated information on Facebook and other privately owned platforms. Furthermore, the very notion of ‘health data’ is changing: with the emergence of big data analytics, any type of data may, in principle, be used as health data if it appears to be associated with health outcomes (Prainsack 2017, p. 70). Such developments may also affect what patients experience as sensitive information and what they see as possible breaches of the tacit norms that guide their expectations on where this information flows (cf. Nissenbaum 2011).

Some health professionals and citizens have recently started to contest the integration and increasing repurposing of patient information. In 2014, a group of GPs—including Bente—mobilized against the mandatory integration of patient information derived automatically from their medical record systems (Wadmann and Hoeyer 2018). In 2019, protests took place over the management of patient data in a newly launched National Genome Center for the shared storage of patients’ genetic information (Kristiansen and Jeppesen 2018; Kjær 2016). In 2021, more than 20,000 Danes opted out of biobank research that relied on national registries of available blood samples—a considerable increase from 2014 when less than 500 people had registred to opt out (Nordfalk 2021). When the COVID-19 pandemic struck, and the government sought to pass a new law on epidemic control, there were public protests against data integration. Klaus Hoeyer has observed groups of citizens taking to the streets in protest against the loss of privacy, as data and test samples were transferred to central databases and reused beyond their own control. In some parts of the populace, confidence in the management of personal information appears to have been shaken.

## Discussion

From the earliest legal documents we have identified, it has been a key concern for lawmakers to establish trust in the handling of patient information. If patients are to feel confident in seeking help, patient information needs protection. Yet ideas about what to protect, and how to do it, have changed considerably over time as patient information has come to take on new forms and been exchanged among still more actors. We have shown how patient information has developed first from orally conveyed stories, to written documentation, and then to digital data. Concurrently, the relationships in which information is exchanged have expanded from patients and named professionals, to include a collective of professionals, government authorities and, lately, international and commercial actors. The early notion of *secrecy* as a social expectation of an unconditional withholding of a personal secret gave way to an emphasis on *confidentiality* understood as a professional duty to exert judgement on when to transfer which information and to whom. Gradually, this notion of confidentiality developed into *informational citizen rights* and rule-based *organizational duties* when health professionals became embedded in a wider societal machinery. Lately, the former ideas and practices of confidentiality have given way to *data protection*. This shift to data protection has turned notions of how to guard information into a technocratic issue. With automated pooling of data, patient information is now administered and protected as a state asset (Pinel and Svendsen 2021). We summarize the findings presented above in Table 2 to convey an overview of the identified developments.

While Denmark has taken a particularly dedicated route towards the integration of information infrastructures (Bauer 2014; Kierkegaard 2013; Hoeyer 2016), we believe that our analysis is relevant beyond the Danish borders. The ambition of establishing integrated data infrastructures in healthcare is international in scope (Wachter 2017), and the challenges involved in the shift in emphasis from confidentiality to data protection also has wider relevance. The drivers of more data-intensive healthcare are likely to vary among contexts. For instance, whereas the development of a state-funded healthcare system played a crucial role for the flow of patient information in Denmark, health insurance seems to have played an equally important role for information generation and exchange formats in the USA during the same period (Koopman et al. 2021). Overall, the Danish history illustrates the basic tension between concurrent ambitions of facilitating information flows while at the same time fostering confidence in sensitive patient information not ending up in unwarranted or unexpected places. This duality has a long legacy (Brown and Duguid 2000) and continues to shape contemporary debates on data regulation (Holm and Ploug 2017; Obar 2017; Taylor et al. 2017).

Even though patient information has come to travel faster and more widely than ever, the social expectation of a confidential relationship between patients and professionals lingers on. Patients still convey sensitive information when they seek help from a health professional, and such a relationship fosters certain expectations. As Simmel reminds us, when people confide in each other, it affects their relationship. It is therefore perhaps not surprising that health professionals continue to discuss data sharing as a problem of interpersonal confidentiality (like Bente does in the opening quote)—even if they now have limited ability to control the flow of patient information. When working on integrated digital platforms, professionals may choose to refrain from datafying particularly sensitive patient information to restrict its flow, but not necessarily control the information entered into the system (Petersson and Backman 2021). Hence, they resort to the former regime of *secrecy* to deal with the technological change. In pace with the political, legal and technological developments outlined in the historical analysis, the idea of confidentiality as a curated transfer of information between people has become obsolete. Even if the term still figures in political and legal documents, it has lost its practical relevance to the regulation of information flows. In short, the reality which the idea of confidentiality once referred to has radically changed. Borrowing from Ulrik Beck, we therefore argue that confidentiality has turned into a ‘zombie category’, i.e. an idea or a concept that lives on even if the reality to which it corresponds has passed away (Beck and Beck-Gernsheim 2002, p. 201–13; Beck and Willms 2004, p. 51–2).

If confidentiality has become a zombie, how may patient interests then be protected? A renewed emphasis on the professional duties of confidentiality does not seem feasible: professionals cannot be held responsible for information flows, which they do not control. The contemporary emphasis on citizens’ rights reflects an empowerment ideal: individual persons are provided with legal rights to better protect their own interests. Yet, the current focus on transparency, portability, erasure and rectification rights in the GDPR does not provide citizens with adequate tools to limit the reuse of their data (Nissenbaum 2011; Obar 2017). Moreover, as information infrastructures grow more complex, it can be difficult for patients and citizens to exert these rights in practice. The informational rights appear to be modelled on a conception of information as contained in physical documents stored in specific and easily identifiable places, which is already outdated. Patient information is increasingly transferred and multiplied in complex digital systems with limited human involvement. Patient information can be in many places at once, and it can be difficult for patients, let alone any health professional, to know where the information is ‘stored’ and what it is used for—and therefore whom to approach with requests about erasure, rectification etc. The old notion of confidentiality as pertaining to a relationship between health professional and patient is losing ground. In its place we have a post-human system of information exchange. The resulting disconnect between social expectations of confidentiality, on the one hand, and the limited ability of professionals to control information flows, on the other, provokes reactions like those of Bente and others who have contested the increasing integration and repurposing of patient data (e.g. Langhoff et al. 2016; Sandvik 2020; Steininger and Stiglbauer 2015; Sterckx et al. 2015; Vezyridis and Timmons 2017; Wadmann and Hoeyer 2018). If data protection does not satify the concerns of patients and health professionals, how then to ensure that the social norms and expectations associated with the conveying of sensitive patient information can be met? Perhaps policymakers first need to see *limitations* to flows as *conducive* to data reuse. Rather than thinking of *access* to data as the main technical and legal problem to be solved, carefully negotiated *restrictions* on access also warrant attention to ensure that data are not used against the interests and expectations of patients. To negotiate limits is a political process, but it is also a process dependent on other types of knowledge that can bring tacit patient norms into the conversation and inform technocratic decisions. To sustain a socially robust ecosystem for health information, it is important for scholars and policymakers alike to acknowledge the interaction of flows and non-flows: the flow of information depends also on the ability to establish and maintain non-flows.

## Appendix 1: Empirical sources informing the historical analysis

**Table:**

Periods	Sources
Before 1930	Forordning om Medicis oc Apotecker og c. (1672). Copenhagen: King Christian V of Denmark
	Kgl. reskript af 13. marts (1750). Copenhagen: King Frederik V of Denmark
	*Kgl. reskript af 6. januar (1764).* Copenhagen: King Frederik V of Denmark
	Kancelliskrivelse af 15. august (1815). Copenhagen: King Frederik VI of Denmark
	Sygekasseloven (1892) LOV nr. 85. Copenhagen: Ministry of Interior
	Instruks for alle til praksis berettigede jordemødre (1896). Copenhagen: Ministry of Interior
	Olrik, E. (1905) Lægers tavshedspligt. *Ugeskrift for Retsvæsen* 1905B: 197–234
	Jordemoderloven (1914). LOV nr. 126. Copenhagen: Ministry of Interior
	Lov om udøvelse af tandlægegerning (1916) LOV nr. 40. Copenhagen: Ministry of Interior
1930–1967	Ministry of Interior (1931) *Betænkning afgivet af Kommissionen Angaaende Lægers Retsstilling.* Copenhagen: Ministry of Interior
	Lov om autoriserede sygeplejersker (1933) LOV nr. 140. Copenhagen: Ministry of Interior
	*Lov om folkeforsikring* (1933) LOV nr. 182. Copenhagen: Ministry of Work and Social Affairs
	Fridericia, H. J. (1934) *Lov om Folkeforsikring. Ændringerne fra Tidligere Lovgivning.* Copenhagen: Levin and Munksgaard
	Lov om udøvelse af lægegerning (1934) LOV nr. 72. Copenhagen: Ministry of Interior
	Bekendtgørelse om lægers pligt til at føre optegnelser (1937) BEK nr. 244. Copenhagen: Ministry of the Interior
	Lov om sygehusvæsenet (1946) LOV nr. 71. Copenhagen: Ministry of Interior
	Backer, K. H. and Skovgaard, A. (1949) LOV nr. 72 af 14. marts 1934 om Udøvelse af Lægegerning med kommentarer og henvisninger. I: K. H. Backer and A. Skovgaard (eds.) *Social-Medicinske Love. Udvalgte og bearbejdede med henblik på deres betydning i social-medicinsk praksis.* Copenhagen: Forlaget for Videnskabelig Litteratur
	NN (1967) *Amtssygehuset eneste hospital uden for USA med en elektronhjerne*, 21 March. Newspaper article retrieved from the local historical achieve of Gentofte
	Registerudvalget (1973) *Delbetænkning om Offentlige Registre* (betænkning nr. 687). Copenhagen: Ministry of Justice
	Ministry of Justice (1976) *Delbetænkning om Offentlige Registre* (betænkning nr. 767). Copenhagen: Statens Trykningskontor
	Ministry of Interior (1977) *Betænkning om grundlaget for en overordnet prioritering af indsatsen inden for sygebehandling og sygdomsforebyggelse* (betænkning nr. 809). Copenhagen: Statens Trykningskontor
	Retsudvalget (1978) *Betænkning over Forslag til lov om offentlige myndigheders registre*. Copenhagen: Ministry of Justice
	Ministry of Justice (1984) *Betænkning om tavshedspligt* (betænkning nr. 998). Copenhagen: Ministry of Justice
	Forvaltningsloven (1985) LOV nr. 571. Copenhagen: Ministry of Justice
	Lov om ændring af lov om private registre m.v. og lov om offentlige myndigheders registre (1987) LOV nr. 383. Copenhagen: Ministry of Justice
	Hollmann, E. 1989. ‘Lægesekretær i 50 år—en kavalkade', *Dansk Lægeskretærforening/HK*: 5–54
	Lov om aktindsigt i helbredsoplysninger (1993) LOV nr. 504. Copenhagen: Ministry of Health
	*Council Directive 95/46/EC on the protection of individuals with regard to the processing of personal data and on the free movement of such data* (1995) Official Journal L281, p. 31
	Patientretstillingsloven (1998) LOV nr. 428. Copenhagen: Ministry of Health
2000-	Council Regulation 2016/976 on the protection of natural persons with regard to the processing of personal data and on the free movement of such data, and repealing Directive 95/46/EC (General Data Protection Regulation) (2016) Official Journal L119, p. 1
	Ministry of Justice (2017a) *Databeskyttelsesforordningen og de retslige rammer for dansk lovgivning* (betænkning nr. 1565). Copenhagen: Ministry of Justice
	Ministry of Justice (2017b) *Forslag til Lov om supplerende bestemmelser til forordning om beskyttelse af fysiske personer i forbindelse med behandling af personoplysninger og om fri udveksling af sådanne oplysninger (databeskyttelsesloven)* (LSF nr. 68)*.* Copenhagen: Ministry of Justice
	Article 29 Working Party (2018) Guidelines on Consent under Regulation 2016/679
	Databeskyttelsesloven (2018) LOV nr. 502. Copenhagen: Ministry of Justice
	European Commission (2020a) *A European Strategy for Data. Communication from the Commission to the European Parliament, the Council, the European Economic and Social Committee and the Committee of the Regions.* Brussels: European Commission
	European Commission (2020b) *White paper on Artificial Intelligence—A European approach to excellence and trust.* Brussels: European Commission
	European Data Protection Board (2020) *Guidelines 05/2020 on consent under* Regulation 2016*/679*. Brussels: European Data Protection Board
	Regeringen (2021) *Strategi for Life Science*. Copenhagen: Ministry of Industry, Business and Financial Affairs

## Footnotes

1 All translations from Danish to English have been made by the authors. Informant names are pseudonyms.

2 The English concept of ‘privacy’ has no direct translation into Scandinavian languages (Hoeyer 2010). In Danish legislation on citizen and patient information, concern for ‘privatlivets fred’, which literally means ‘undisturbed private-life’, is typically mentioned but not given particular emphasis (e.g. Ministry of Justice 1976).

3 Note that regulation centers specifically on ‘data’ from the late 1970s, whereas the management of patient information was regulated through legislation that focussed on the duties of ‘professionals’ in the previous period.

4 See e.g. Recommendation 509 to the Committee of Ministers in 1968: https://assembly.coe.int/nw/xml/XRef/Xref-XML2HTML-en.asp?fileid=14546&lang=en, Europarådets Resolution (73) 22, Europarådets Resolution (74) 29.
